# Lingering challenges in malaria elimination efforts in sub‐Saharan Africa: Insights and potential solutions

**DOI:** 10.1002/hsr2.2122

**Published:** 2024-06-02

**Authors:** Lukman Lawal, Ahmad Oyindamola Buhari, Tawakalitu Abdulateef Jaji, Abdulrahman Salaudeen Alatare, Abdulmalik Opeyemi Adeyemo, Aishat Oluwakemi Olumoh, Yusuff Adesoji Yusuff, Gabriel Osborn, Abdulazeez Biodun Mogaji, Bello Hussein Adoto, Nafisa Gbemisola Ibrahim, Waliyullahi Oluwafemi Saliu, Toufik Abdul‐Rahman

**Affiliations:** ^1^ Centre for Malaria and Other Tropical Diseases Ilorin Nigeria; ^2^ Faculty of Clinical Sciences University of Ilorin Ilorin Nigeria; ^3^ Medical Institute Sumy State University Sumy Ukraine; ^4^ Toufik's World Medical Association Sumy Ukraine

**Keywords:** artemisinin combination therapy, insecticide treated net, malaria control and elimination, Plasmodium spp, R21/Matrix‐MTM malaria vaccine, RTS, S, sub‐Saharan Africa, world malaria report

## Abstract

**Introduction:**

Between 2000 and 2015, significant gains were recorded in reducing the global burden of malaria due to enhanced global collaboration and increased funding. However, progress has stagnated post‐2015, and the COVID‐19 pandemic seems to have reversed some of these gains, necessitating a critical reevaluation of interventions. This paper aims to analyze the setbacks and offer recommendations for advancement in malaria control and prevention in sub‐Saharan Africa.

**Methods:**

We conducted searches on Google Scholar, PubMed, and relevant organization websites to identify relevant studies on malaria control and prevention and associated challenges in sub‐Saharan Africa from 2015 to the present. Additionally, studies on individual sub‐Saharan African countries were reviewed to ensure comprehensiveness. Data from selected studies were extracted and analyzed using a narrative synthesis approach to offer a concise overview of the evidence.

**Findings:**

We observe that the halt in progress of malaria control in sub‐Saharan Africa has deep roots in socioeconomic, political, and environmental factors. These challenges are exacerbated by the population explosion in the region, low coverage of interventions due to funding deficits and incessant crises, and the degradation of the efficacy of existing malaria commodities.

**Conclusion:**

Sub‐Saharan Africa is at a crossroads in its fight against malaria. Promising new frontiers such as malaria vaccines, preventive monoclonal antibodies, new‐generation insecticide‐treated nets, and potentially artificial intelligence‐driven technologies offer hope in advancing malaria control and prevention in the region. Through commitment and collaboration, leveraging these opportunities can help surmount challenges and ultimately eliminate malaria in sub‐Saharan Africa.

## 1 INTRODUCTION

The buzzing of mosquitoes is an ever‐present reminder to many in sub‐Saharan Africa (SSA) of the imminent threat of malaria. Malaria, caused by the mosquito‐borne Plasmodium parasite, is a longstanding affliction in tropical regions, with the greatest burden in SSA.^1^

Between 2000 and 2015, unprecedented success against malaria was achieved because of renewed engagement among global stakeholders. The establishment of large international funders such as the Bill & Melinda Gates Foundation in 2000, the Global Fund in 2002, and the US President's Malaria Initiative in 2005 significantly increased funding for malaria research, development, and implementation programs, leading to the World Health Organization's approval of the first artemisinin combination therapies (ACTs), malaria rapid diagnostic tests (RDTs), and long‐lasting insecticide‐treated nets (LLITNs). Other prominent control strategies include indoor residual spraying (IRS), personal protection, and malaria chemoprevention. These strategies revolve around the guidelines proposed in the global technical strategy for malaria 2016–2030, aiming to achieve a 90% reduction in incidence and mortality rates by 2030 as compared to 2015.^2^

However, despite these efforts, the falling trend in malaria rates in SSA has plateaued since 2015, and we are now facing a reversal of the modest gains.^2^ Sub‐Saharan Africa continues to tackle a host of challenges limiting its eradication efforts, which was exacerbated by the COVID‐19 pandemic.^3^ Of the 247 million malaria cases and 619,000 deaths reported worldwide in 2021, SSA accounted for 95%, with children under 5 and pregnant women contributing >80% of the mortality. The setbacks in meeting the 2020 malaria control targets have now warranted a reassessment of existing interventions and strategies. Therefore, this article seeks to both analyze the encountered difficulties and suggest tailored suggestions to address them.

## 2 METHODS

We conducted searches on Google Scholar, PubMed, and relevant organization websites to identify relevant studies on malaria control and prevention and associated challenges in sub‐Saharan Africa from 2015 to the present. The search terms used were “malaria,” “control or prevention” and “sub‐Saharan Africa.” Additionally, studies on individual sub‐Saharan African countries were reviewed to ensure comprehensiveness. Data from selected studies were extracted and analyzed using a narrative synthesis approach to offer a concise overview of the evidence.

## 3 FINDINGS

### 3.1 Current measures of malaria control and prevention

The current eradication strategies, which employ a tailored approach introduced in 2018, consider multiple determinants to optimize resource utilization. The pillars of this approach are: ensuring prevention through vector‐control methods (such as IRS and the use of LLITN), chemoprevention in select groups, diagnostic testing (RDT and microscopy), and treatment with ACT; accelerating efforts to attain malaria‐free status by targeting transmission foci, facilitated by active case detection and investigation; and weaponizing surveillance by augmenting national malaria programs with health information systems that aid in resource reallocation, identifying gaps, detecting outbreaks, and assessing the impact of interventions.^2^

Despite the effective strategies and investments made by nations, progress toward zero malaria has largely stalled, with only 9 out of 47 WHO‐African countries meeting the 2020 targets.^1^

### 3.2 The challenges associated with malaria control and prevention in SSA

Malaria is a complicated problem in SSA with deep roots in socioeconomic, political, and environmental factors. High transmission rates are caused by the tropical climate and a bad drainage system. Furthermore, rapid population growth made more people susceptible to malaria and decreased investment per person. The non‐resilient health system in sub‐Saharan Africa compromises the effectiveness and timeliness of malaria diagnosis and treatment. Poor disease surveillance, an insufficient workforce, poor provider adherence to clinical protocols, poor patient accessibility, and the affordability of proper healthcare due to poverty leading to the patronage of unconventional care providers are all factors that need to be addressed.^4^

Additionally, inadequate intervention coverage hinders the eradication of malaria in sub‐Saharan Africa, as fewer than 25% of children sleep under an ITN in some SSA countries. Only 38% of those who sought medical attention were tested for parasites, of which only 57% received the recommended anti‐malarial therapy (ACT) in 2021, and only about 25% of pregnant women received the intermittent preventive therapy doses between 2015 and 2021.^3^ This exemplifies how disparities in healthcare access and under‐utilization of the interventions limit the effectiveness of these interventions.

Also, ongoing efforts and research are hampered by ineffective government policies and funding. Only 50% of the estimated US$ 7.3 billion needed globally in 2021 to stay on track to defeat malaria was raised.^1^ The funding shortfall made the economic downturn brought on by high‐impact crises like the COVID‐19 pandemic, epidemics, and ongoing conflicts in many SSA nations—that disrupt supply, production, and funding chains—even worse.^5^, ^6^, ^7^

Another major barrier is the gradual degradation of the efficacy and effectiveness of antimalarial drugs and other malaria‐related products. The diversity of malaria vectors, the expanding emergence of Anopheles stephensi in SSA, the inaccuracy of PfHRP‐2‐based RDT kits due to PfHRP‐2 deletion, and vector insecticide and IRS resistance are all significant headwinds that must be overcome to advance the eradication of malaria in SSA.^8^, ^9^, ^10^, ^11^ Arguably, malaria vaccines may offer fresh hope, but vaccine hesitancy, as evidenced by the COVID‐19 pandemic, poses a serious threat.^12^

### 3.3 Potential solutions

The salient lessons lie in the success stories of other countries in eradicating malaria. China demonstrated the potency of existing measures by achieving malaria‐free status after 4 years of zero cases. This feat was largely attributed to adequate government funding and strict adherence to established protocols for widespread testing and distribution of vector control apparatus, with progress maintained through tight surveillance and prompt response to reported cases.^13^, ^14^ The success provides valuable insights for countries grappling with the challenges of malaria eradication. It is high time relevant stakeholders invest wisely, innovate smartly, and implement precisely to deliver zero malaria in SSA.

**FIGURE 1:**
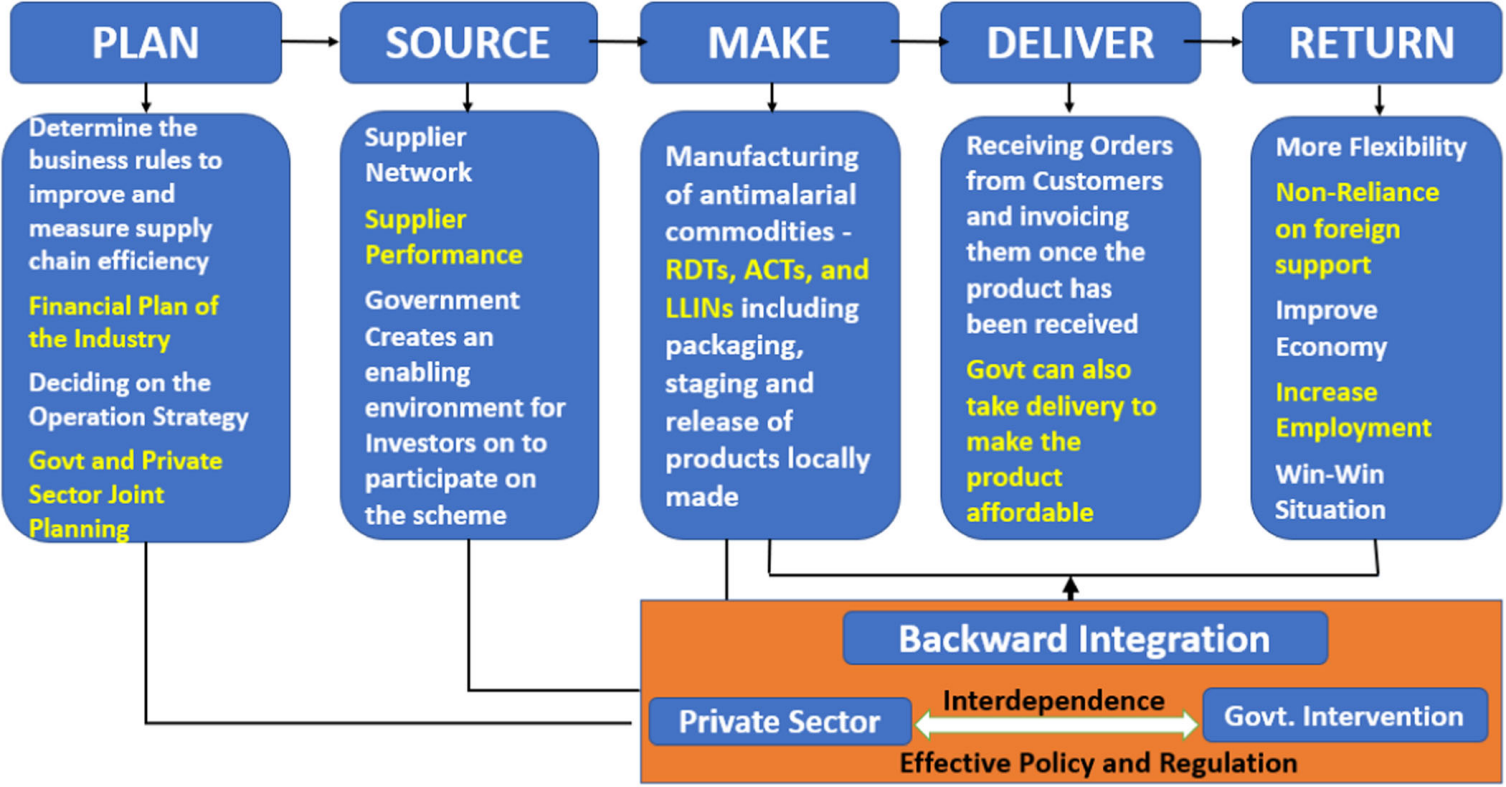
Environmental management processes for backward integration approach. *Credit*: Mokuolu et al.^15^

The current funding deficit is projected to widen as funding needed to reach the global malaria control target is estimated to increase to US$ 9.3 billion in 2025.^2^ As a result, high‐level political commitment must translate into predictable and long‐term financing for malaria programs. In addition to increasing domestic and international funding, alternative innovative funding mechanisms, such as backward integration through a Government‐led public–private partnership should be considered to expand the available resources. Figure 1 illustrates the framework and processes for the backward integration approach.^15^ Adopting the backward integration funding mechanism will allow for the sustainability of the malaria control program as the supply chain including production, distribution, logistics, and design would be under the control of the Government in partnership with the private sector.^15^ This will enhance local production of malaria intervention commodities that are accessible and affordable using market‐based demand and supply arrangements. The other promising funding mechanisms include the Drug Revolving Fund, the National Health Insurance Scheme, and the Malaria Tax. Adequate funding will ensure that malaria services are accessible and affordable to the most vulnerable populations, wherever they are, thereby also expanding the coverage of intervention.

It is also important that all SSA countries implement the High Burden to High Impact concept to break down barriers and reach the underserved and most vulnerable groups. Improving technical capacity and leadership for public health practice and research will aid in improving data quality and data use to support national and subnational malaria responses.^4^

Furthermore, adopting novel approaches to surmount roadblocks to malaria eradication and deliver successful interventions is necessary to advance malaria control. The artificial intelligence (AI) driven microscope has already shown that it can identify malaria parasites with a degree of precision that complies with WHO microscopy standards. AI‐based microscopes may be especially useful in identifying drug‐resistant parasite strains by classifying drug‐induced morphological outliers according to the mode of action and detecting low‐level parasitemia using a deep learning‐based analysis after staining with 4′,6‐diamidino‐2‐phenylindole fluorogen.^16^ Additionally, AI, utilizing either structure‐based or ligand‐based approaches, has shown highly accurate results in the field of chemical property prediction. Therefore, AI would be a suitable alternative to the traditional drug discovery approach of high‐throughput screening, which is time‐consuming and resource‐intensive, in the discovery of novel antimalarials to combat the emerging artemisinin‐resistant plasmodium falciparum.^17^ AI‐powered diagnostic panels could also be implemented to differentiate between viral, bacterial, and malaria infections, which will help enhance clinical care for patients. In addressing environmental drivers, AI could be used to analyze climatic data to identify areas at risk of seasonal outbreaks of malaria and provide focused preventative measures.

Regarding vector resistance, it can be tackled by expanding the distribution of new‐generation, long‐lasting ITNs. The WHO now recommends the use of two new ITNs: Pyrethroid‐chlorfenapyr nets, which combine a pyrethroid and a pyrrole insecticide to enhance the killing effect of the net, and Pyrethroid‐pyriproxyfen nets, which combine a pyrethroid with an insect growth regulator, which disrupts mosquito growth and reproduction. In comparison to pyrethroid‐only nets or pyrethroid‐PBO nets, the new generation nets have a greater impact against malaria vectors, and ultimately, on malaria.^18^ However, the cost of full coverage of distribution can be prohibitive in resource‐poor settings. Therefore, it is strategically desired that the main objective is to attain comprehensive coverage in specific areas by providing pyrethroid‐only insecticide‐treated nets (ITNs) and determining the required funding. When extra funds are available, the approach changes to improve effectiveness. This includes replacing pyrethroid‐only ITNs with pyrethroid‐PBO or pyrethroid‐chlorfenapyr ITNs in regions where pyrethroid resistance is a concern. It also involves replacing previously distributed nets in affected zones and expanding their deployment to other areas based on decreasing malaria risk. The vital step of identifying funding gaps that impede effective coverage is essential and should be communicated to potential donors.^18^

Targeted indoor residual spraying (TIRS) is another innovative approach that focuses on applying insecticides to specific indoor areas where mosquitoes rest.^18^ Unlike traditional spraying methods, TIRS minimizes environmental impact while effectively reducing mosquito populations and malaria transmission. This targeted strategy enhances the efficiency of vector control efforts, providing a more sustainable and eco‐friendly approach to malaria prevention.

Moreover, clustered Regularly Interspaced Short Palindromic Repeats technology also offers a potential solution for vector control by allowing gene drives to repopulate endemic regions with genetically modified mosquitoes resistant to the Plasmodium parasite, although ethical concerns need to be addressed before its use can be considered.^19^ Gene drive technology, a groundbreaking approach, involves altering mosquito genetics to disrupt malaria transmission by reducing their ability to transmit the parasite.^20^ Similarly, Wolbachia‐based strategies introduce bacteria into mosquito populations, reducing their vector competence for malaria parasites and potentially curbing disease transmission.^21^ These genetic interventions represent innovative tools in the fight against malaria, offering targeted solutions to control mosquito populations and reduce disease burden.

Chemoprevention could be advanced by scaling up community intermittent preventive therapy in pregnancy uptake via community engagement in management, studying the climatic information of SSA countries, expanding seasonal malaria chemoprevention where appropriate,^22^ and systematically deploying malaria vaccination as an additional tool.^23^ The WHO‐recommended malaria vaccine (RTS,S) and the R21/Matrix‐M are potential game changers in the battle to end malaria.^24^ Vaccination complements existing interventions by providing additional protection against the malaria parasite, particularly in high‐transmission areas. The concern of vaccine hesitancy, as seen with the Covid‐19 vaccine, can be addressed by identifying gaps in knowledge and information dissemination about malaria vaccines as well as understanding the factors that could influence vaccine uptake. This will help tailor effective strategies for the introduction and promotion of malaria vaccines in the region.

Moreover, the preventive monoclonal antibody, CIS43LS, which is safe and effective in African adults, reinforces the renewed hope, as it potentially complements current chemopreventive measures. In the phase two clinical trial featured in the New England Journal, researchers evaluated the effectiveness of CIS43LS in two ways. In terms of the time it took for the first P. falciparum infection to occur over the 24‐week study period.^25^ In terms of the time it took for the first P. falciparum infection to occur over the 24‐week study period, the high dosage (40 mg/kg) of CIS43LS exhibited an 88.2% efficacy in preventing infection, while the lower dosage (10 mg/kg) demonstrated a 75% effectiveness. When analyzing the percentage of participants infected with P. falciparum at any point during the 24‐week study, the high dosage was found to be 76.7% effective, and the lower dosage was 54.2% effective. These initial field results indicate that a monoclonal antibody can safely offer substantial protection against intense malaria transmission in healthy adults. As a result, further investigations are recommended to assess whether such an intervention can prevent malaria infection in infants, children, and pregnant women.^25^

Turning to Artemisinin resistance, it could be addressed by educating healthcare providers, including patent and proprietary medicine vendors and community extension workers, and the general public on the importance of testing before treatment and treatment adherence while promoting the systematic use of various ACTs through education, multi‐level policymaking (adding different ACTs to the essential drug list), and a review of treatment guidelines emphasizing the use of various ACTs. This will help reduce the indiscriminate use of the Artemether‐Lumefantrine combination, thereby reducing the risk of artemisinin resistance. More so, political will must translate into more resources and actions needed to expedite the completion of the investigation of retooled antimalarials such as triple ACT and newer drugs, such as KAE609 (Cipargamin), KAF156 (Ganaplacide) + Lumefantrine, DSM265 and Oz439 (Ferroquine) for possible approval for use.^26^

Overall, several factors support the feasibility of malaria eradication in SSA. Scientific advances, such as improved diagnostics, novel vector control methods, and the development of malaria vaccines, offer promising tools for malaria control and elimination. Through targeted education campaigns involving mothers, caregivers, community health workers, and leaders, communities can be empowered with knowledge about preventive measures such as the use of insecticide‐treated bed nets (ITNs), indoor residual spraying, proper diagnosis, and prompt treatment. These interventions can lead to reduced malaria prevalence and mortality in pregnant women and children under 5.^27^, ^28^, ^29^ Implementing integrated approaches that combine vector control, case management, surveillance, and community mobilization can maximize impact and accelerate progress toward eradication.

## 4 CONCLUSION

The fight against malaria in SSA is at a critical juncture. While effective eradication methods exist, obstacles impede their efficacy, jeopardizing progress. Achieving the ambitious goals of the GTS for Malaria (2030) necessitates judicious resource allocation, innovative approaches, and precise interventions. Promising new frontiers such as malaria vaccines, preventive monoclonal antibodies, new‐generation ITNs, and potentially AI‐driven technologies offer hope in advancing malaria control and prevention in the region. Through commitment and collaboration, leveraging these opportunities can help surmount challenges and ultimately eliminate malaria in SSA.

## AUTHOR CONTRIBUTIONS

**Lukman Lawal**: Conceptualization; writing—original draft; writing—review & editing. **Ahmad Oyindamola Buhari, Tawakalitu Abdulateef Jaji, Abdulrahman Salaudeen Alatare, Abdulmalik Opeyemi Adeyemo, Aishat Oluwakemi Olumoh, Yusuff Adesoji Yusuff, Gabriel Osborn, Abdulazeez Biodun Mogaji, Bello Hussein Adoto, Nafisa Gbemisola Ibrahim**, and **Waliyullahi Oluwafemi Saliu**: Conceptualization; writing—original draft. **Toufik Abdul‐Rahman**: Writing—review & editing.

## CONFLICT OF INTEREST STATEMENT

The authors declare no conflict of interest.

## ETHICS STATEMENT

Ethics approval was waived for this study because no patients' data were reported.

## TRANSPARENCY STATEMENT

The lead author, Toufik Abdul‐Rahman, affirms that this manuscript is an honest, accurate, and transparent account of the study being reported; that no important aspects of the study have been omitted; and that any discrepancies from the study as planned (and, if relevant, registered) have been explained.

## Data Availability

Data sharing is not applicable to this article as no new data were created or analyzed in this study.
